# Detection of rotational errors in single‐isocenter multiple‐target radiosurgery: Is a routine off‐axis Winston–Lutz test necessary?

**DOI:** 10.1002/acm2.13665

**Published:** 2022-06-17

**Authors:** Lauren M. M. Pudsey, Giordano Biasi, Anna Ralston, Anatoly Rosenfeld, Joel Poder

**Affiliations:** ^1^ Centre for Medical Radiation Physics, School of Physics University of Wollongong Wollongong New South Wales Australia; ^2^ Peter MacCallum Cancer Centre Melbourne Victoria Australia; ^3^ St George Hospital Cancer Care Centre Kogarah New South Wales Australia

**Keywords:** off‐axis Winston–Lutz, single‐isocenter multiple‐target, stereotactic radiosurgery

## Abstract

**Purpose:**

Recently the use of linear accelerator (linac)‐based stereotactic radiosurgery (SRS) has increased, including single‐isocenter multiple‐target SRS. The workload of medical physicists has grown as a result and so has the necessity of maximizing the efficiency of quality assurance (QA). This study aimed to determine if measurement‐based patient‐specific QA with a high‐spatial‐resolution dosimeter is sensitive to rotational errors, potentially reducing the need for routine off‐axis Winston–Lutz (WL) testing.

**Methods:**

The impact of rotational errors along gantry, couch, and collimator axes on dose coverage of the gross tumor volume (GTV) and planning target volume (PTV) was determined with a 1‐mm GTV/PTV expansion margin. Two techniques, the off‐axis WL test using the StereoPHAN MultiMet‐WL Cube (Sun Nuclear Corporation, Melbourne, Florida, USA) and patient‐specific QA using the SRS MapCHECK (Sun Nuclear Corporation, Melbourne, Florida, USA), were assessed on their ability to detect introduced errors before target coverage was compromised. These findings were also considered in the context of routine machine QA of rotational axis calibrations.

**Results:**

Rotational errors significantly impacted PTV dose coverage, especially in the couch angle. GTV dose coverage remained unaffected except for with large couch angle errors (≥1.5°). The off‐axis WL test was shown to be sensitive to rotational errors with results consistently exceeding tolerance levels when or before coverage fell below departmentally accepted limits. Although patient‐specific QA using the SRS MapCHECK was previously validated for SRS, this study showed inconsistency in detection of rotational errors.

**Conclusions:**

It is recommended that off‐axis WL testing be conducted regularly to supplement routine monthly machine QA, as it is sensitive to errors that patient‐specific QA may not detect. This frequency should be determined by individual departments, with consideration of GTV–PTV margins used, limitations on target off‐axis distances, and routine mechanical QA results for particular linacs.

## 1 INTRODUCTION

The increasing use of stereotactic radiosurgery (SRS), including single‐isocenter multiple‐target (SIMT) SRS,^1^, ^2^, ^3^, ^4^, ^5^, ^6^ has increased the workload for medical physicists. Therefore, improving the efficiency of quality assurance (QA) has become progressively more important.

Due to the ablative doses delivered to small targets in SRS, accuracy in all aspects of delivery is essential. Traditionally, linac‐based SRS treated multiple targets individually with the machine isocenter aligned to each target center.^7^, ^8^, ^9^, ^10^ In this scenario, rotational errors have been shown to have minimal effect.^11^ In recent years, there has been growing interest in SIMT SRS,^7^, ^8^, ^10^, ^12^, ^13^, ^14^, ^15^ in which the machine isocenter is usually positioned at the center of the target distribution,^7^, ^10^ that is, a single isocenter is positioned at a point between a group of targets to be treated simultaneously. In this case, the targets are no longer located at the machine isocenter, but at off‐axis positions that will be from herein referred to as off‐axis targets. As a result, rotational errors will have a translational effect upon the dose map with reference to target positions. Previous studies have shown that rotational errors may have a significant impact on gross tumor volume (GTV) and planning target volume (PTV) coverage in SIMT SRS.^16^, ^17^, ^18^ Nakano et al.^17^ investigated the maximum off‐axis distance to preserve 95% coverage of various sized GTVs when using a 1‐mm GTV–PTV expansion. At a 0.5° rotational error, the maximum distance with acceptable coverage of the smallest GTV (1.0‐cm diameter) was 7.6 cm. A study was conducted by Prentou et al.^16^ on the impact of rotational errors on target coverage and other parameters, including conformity index, *D*95%, and dose to organs at risk. For some plans, it was found that even the smallest tested rotation of 0.5° resulted in unacceptable plans. Therefore, verification of not only the spatial distribution/location but also the rotational alignment of the high‐dose region(s) is an important part of SRS QA.

The off‐axis Winston–Lutz (WL) test assesses the mechanical alignment of targets on a medical linear accelerator (linac) to planned radiation high‐dose regions at off‐axis positions. The mechanical isocenter of a linac is at the intersection of the three primary axes of rotation: the gantry, the couch, and the collimator.^19^ The radiation isocenter is the intersection of radiation beams delivered at varied gantry, couch, and collimator angles.^20^ Inaccuracy in locating the mechanical isocenter and aligning this to the radiation isocenter can result in a mismatch between planned and delivered dose distribution.^21^ Inaccuracies may occur as a result of factors such as gantry sag or MLC leaf‐bank sag throughout rotation due to gravity,^22^ misalignment of axes,^22^ linac head imbalance, and wear and tear of bearings.^21^, ^23^ As SRS is often delivered with multiple noncoplanar arcs, alignment of the couch rotational axis must also be verified in addition to the gantry and collimator axes.^21^, ^23^ Presented by Lutz et al.^19^ in 1988, the WL test is a method of verification of the machine isocenter position. The main concept of the WL test remains unchanged; however, advancements in technology have led to modified methodologies to improve accuracy and efficiency resulting in variations of the original WL test. For modern SRS deliveries, the imaging isocenter must also be considered, with the implementation of image‐guided radiotherapy, patient positioning has become less reliant on lasers and instead commonly utilizes onboard imaging.^24^

In 2016, Gao et al.^13^ developed the WL–Gao test that involves positioning a phantom containing a metal ball bearing at several off‐axis distances. Images of the ball bearing are then captured at eight angle combinations in each off‐axis distance and the inaccuracy of the off‐axis target position in relation to the intersection of radiation beams is analyzed. This test was used to determine the distance from the isocenter that a target can be positioned and have acceptable accuracy for that particular linac. This test has since been further developed,^25^ and, in addition, several purpose made QA phantoms for SIMT SRS have been developed which include functionality for off‐axis WL testing.^12^, ^26^ Commercially, the StereoPHAN MultiMet‐WL Cube (Sun Nuclear Corporation, Melbourne, Florida, USA) is now available containing five ball bearings at various off‐axis positions and one at isocenter.

This study assessed the sensitivity in determining off‐axis target misalignment using two methods: (1) off‐axis WL test; and (2) measurement‐based patient‐specific QA with the SRS MapCHECK (Sun Nuclear Corporation, Melbourne, Florida, USA). Patient‐specific QA compares a measured dose distribution to a planned dose distribution and, hence, may detect various delivery errors in a single QA test, including dose miscalculation, MLC leaf errors, and a dosimetric shift, as a result of rotational or translational misalignment. If findings show that patient‐specific QA is as sensitive to rotational errors as the off‐axis WL test, then the latter may be required less frequently as these errors will be detected by the patient‐specific QA already in place. Otherwise, the necessity of a frequent off‐axis WL test would be confirmed as currently there are no guidelines regarding this testing frequency. Analyses of findings also took into consideration routine machine QA that includes gantry, collimator, and couch rotational axis calibrations.

## 2 METHODS

Plans were delivered using a TrueBeam linac (v2.7, Varian Medical Systems, Palo Alto, CA) with a Millennium MLC. Errors in the alignment of off‐axis targets and corresponding off‐axis high‐dose regions were intentionally introduced via rotations in gantry, couch, and collimator angles in the DICOM plan file. The magnitude of rotational error (°) at which coverage fell outside acceptable limits (>99% of the target receiving 100% of the prescription dose) for GTVs and PTVs was determined via the dose–volume histogram analysis. The two techniques, the off‐axis WL test and measurement‐based patient‐specific QA, were assessed on their ability to detect the introduced errors before target coverage was compromised.

In addition, the stability of rotational axes calibration results was analyzed, as measured during routine monthly QA for three available TrueBeam linacs.

### 2.1 Routine monthly mechanical axes testing

The following tests for gantry, collimator, and couch angle indicators were carried out monthly as per routine QA.

Absolute zero for the gantry angle was determined using a digital spirit level held against the flat reference collimator surface. The gantry angle at which the digital spirit level recorded an angle of 0.0° was recorded as absolute zero for the gantry angle.

In order to find absolute zero for the collimator angle, first, the collimator was set to 0° on the digital reading and the field size set to 40 × 40 cm^2^. The floor was marked along the transverse crosshair with the gantry set to be at an angle of approximately 0°. The gantry was then rotated back and forth, and any movement from the marked crosshair position was observed. The collimator angle was adjusted until there was no movement from the marked position; it was at this position that absolute zero for collimator angle was recorded.

In order to verify couch position, gantry and collimator angles were first set to their absolute zero positions, the field size to 40 × 40 cm^2^, and the couch to 0° from the digital reading. The couch pitch and roll were also set to 0° and tape was placed on the couch and the radial crosshair position was marked. The couch was then moved longitudinally and any deviations from the markings were observed. The couch angle was adjusted until deviation was minimized and the digital couch angle reading was recorded as the absolute zero value for the couch angle.

### 2.2 Off‐axis Winston–Lutz testing

**FIGURE 1:**
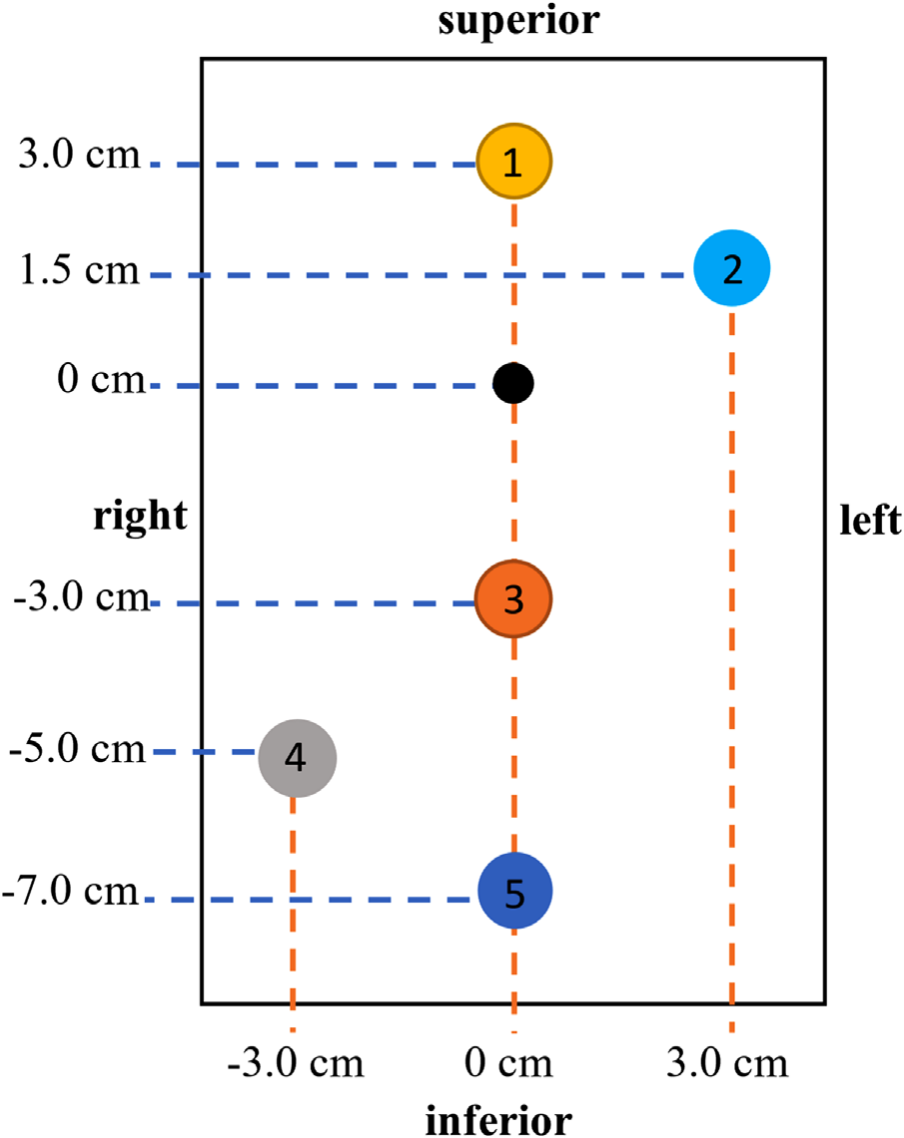
Top‐down view schematic diagram of the ball‐bearing positions within the MultiMet‐WL Cube. These positions also correspond to PTV locations relative to isocenter, numbered 1–5. PTV, planning target volume; WL, Winston–Lutz

The off‐axis WL test was conducted using the StereoPHAN MultiMet‐WL Cube. The cube itself is an 8.5 × 8.5 × 12.75‐cm^3^ acrylic block containing six tungsten carbide spheres of diameter 5.000 ± 0.025 mm, in a central plane at locations outlined in Figure 1. The machine isocenter was assigned to the [0, 0, 0] coordinate of the phantom and the other targets numbered 1–5 from superior to inferior.

**FIGURE 2:**
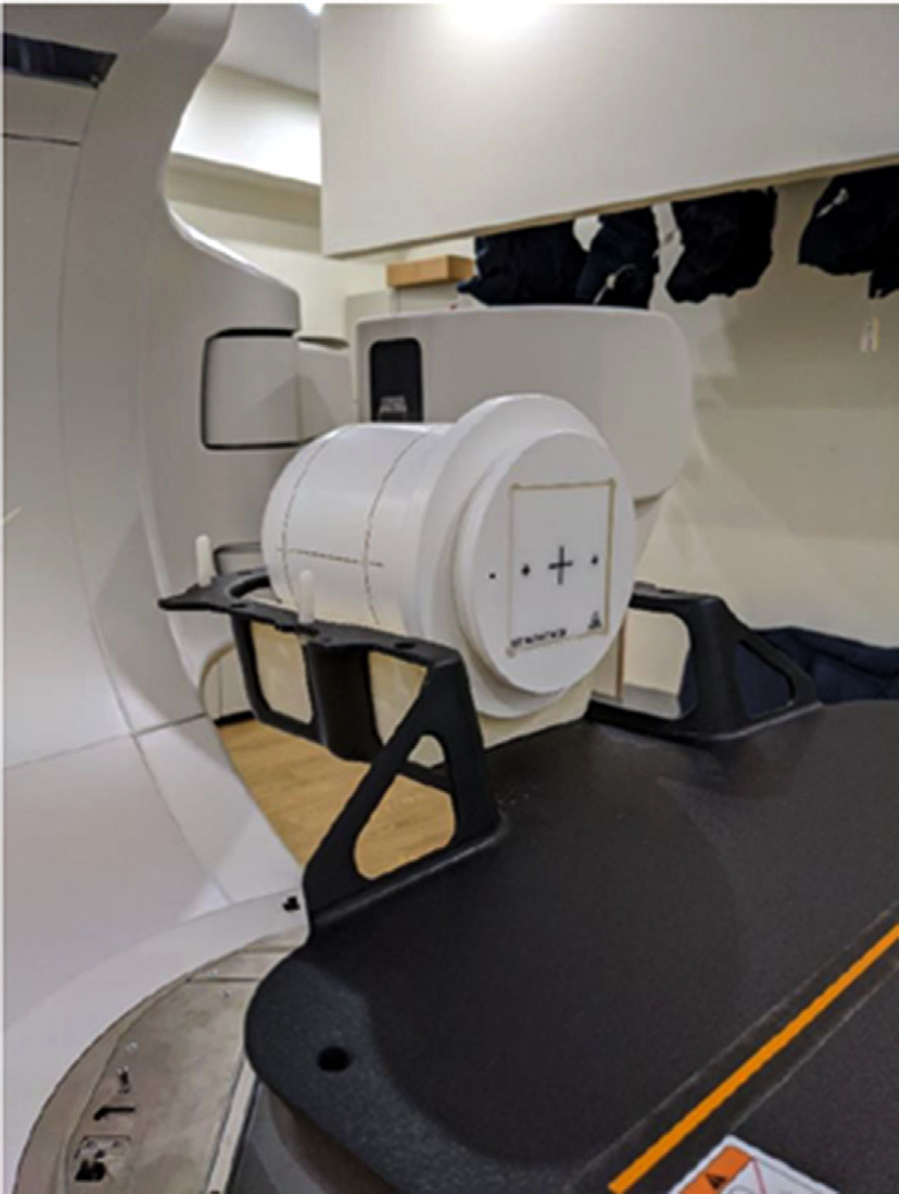
MultiMet‐WL Cube inserted within the StereoPHAN, and positioned on the QFix Encompass SRS headboard. SRS, stereotactic radiosurgery; WL, Winston–Lutz

The cube was inserted into the StereoPHAN, then positioned on the treatment couch using a foam cradle shaped to fit the phantom within the QFix Encompass SRS headboard (Type RT‐4600‐01, QFix, Avondale, PA) (Figure 2). The phantom was aligned to the machine isocenter using the room lasers and surface markers. A cone‐beam CT image of the setup with a 1‐mm slice thickness was obtained and a six degrees of freedom PerfectPitch couch was used to align the acquired image to a previously obtained reference CT image, also with a 1‐mm slice thickness.

Images of the ball‐bearing positions were obtained using the electronic portal imaging device and an Eclipse (v13.6, Varian Medical Systems, Palo Alto, CA) treatment plan supplied with the MultiMet‐WL from Sun Nuclear Corporation. The plan irradiated each target with an MLC‐defined 2 × 2‐cm^2^ beam at eight combinations of gantry, couch, and collimator angles, as outlined in Table 1. When coplanar target positioning occurred at gantry angles 90° and 270°, two images were obtained of a subset of targets to avoid target overlap.

**TABLE 1:** Gantry, couch and collimator angle combinations at which images of the targets were captured

Gantry angle (°)	Couch angle (°)	Collimator angle (°)
180	0	90
90	0	90
270	0	90
0	0	90
0	90	0
0	270	0
0	0	270
0	0	0

Errors in gantry, couch, and collimator angles were introduced one at a time by manual entry into the delivery system at values between 0.0° and 2.0° in increments of 0.5° away from their absolute zero values. These rotations were only in the positive direction as the field delivered was uniform and the phantom was accurately aligned with image guidance, therefore, being a geometric issue, rotations in the opposite direction would comprise unnecessary repetition.

Results of the off‐axis WL test were analyzed using the MultiMet‐WL Analysis tool (v1.0, Sun Nuclear Corporation, Melbourne, Florida, USA). The set of obtained DICOM images for each test was accessed by the analyzer that determines the positioning error for each target. The analyzer automatically recognizes images based upon information in the DICOM files, including gantry, couch, and collimator angle. Therefore, in order for the files with errors introduced to be analyzed, the DICOM files had to be edited to contain the original angles. This was performed via a script using the Python programming language (v.3.5.8, Python Software Foundation, Fredericksburg, Virginia, USA) and compiled using Conda (v.4.9.2, Anaconda Software Distribution, Austin, Texas, USA). The Python package Pydicom (v.2.1.2) was used to read in the DICOM files and the angles were corrected.

Each of the targets was analyzed separately with the discrepancy between actual and expected target positions assessed for (1) the *x*‐ and *y*‐direction in each image, (2) the 2D position in each image, and (3) the discrepancy in 3D position calculated using all eight images. For the results of the test to be acceptable, discrepancies in all of the abovementioned factors must be <1.00 mm.

### 2.3 GTV and PTV dose coverage

An SIMT SRS treatment plan was created based on the location of the targets in the MultiMet‐WL phantom using four volumetric modulated arc therapy beams with couch angles at 0, 45, 90, and 315°, as shown by Clark et al.,^27^ to produce high‐quality SRS plans. The plan was created in the Varian Eclipse treatment planning system (TPS) with a prescription of 20 Gy in one fraction to five 1‐cm diameter spherical PTVs with a 1‐mm GTV–PTV margin (i.e., GTV diameter of 8 mm). A 1‐cm diameter PTV was chosen as it is the representative of the average target size in SRS patients treated in our department, and a 1‐mm margin is used in line with our departmental policy for these patients. The isocenter was placed at coordinate 0, 0, 0 in the MultiMet‐WL phantom (Figure 1).

The plan was optimized with jaw tracking turned off and the normal tissue objective manually set at values in Table 2. A normal brain control region was created that is the brain volume minus each of the PTV volumes referred to as “brain‐PTV.” In addition, several ring‐shaped control regions were used (inner, middle, and outer). The “inner control region” is the volume outside the PTV margin and inside a boundary at PTV margin +0.5 cm. The “middle control region” is the volume outside the inner control region and inside a boundary at PTV margin +1.0 cm. The “outer control region” is a volume outside the middle control region and inside a boundary at PTV margin +3.0 cm. The optimization objectives used are outlined in Table 3. Dose was calculated for this plan with a 1‐mm dose grid resolution and without any errors introduced, hereby referred to as the baseline plan.

**TABLE 2:** Normal tissue objective manual settings used

**Parameter**	**Value**
Priority	100
Distance	0.10 cm
Start	100%
End	30%
Falloff	0.50

**TABLE 3:** Optimization objective functions used in treatment planning

**Control Region**	**Limit type**	**Volume (%)**	**Dose (%)**	**Priority**
PTVs	Upper	0.0	140	100
	Lower	100	100	130

**Control Region**	**Limit type**	**Dose**	**Priority**	**α**
Brain‐PTV	Upper gEUD	1.00 Gy	60	1
Inner	Upper gEUD	97%	70	1
Middle	Upper gEUD	50%	70	1
Outer	Upper gEUD	40%	70	1

Abbreviation: PTVs, planning target volumes.

The baseline plan was replicated, and dose was recalculated (without re‐optimization) with rotations in gantry, collimator, and couch angles of 0.0–2.0° in 0.5° intervals. This was done in both positive and negative directions as the off‐axis dose distribution within the high‐dose region was not uniform and, therefore, effects may be asymmetrical. The collimator and couch angles were changed manually within the TPS. However, in order to change the gantry angles, the plan DICOM files were altered using a Python script in Conda and then reimported to the TPS. Upon recalculation, the coverage of each PTV with the 100% isodose line (PTV V100%) was observed to determine if it was still within departmental tolerance, for example, PTV V100% > 99%. Coverage of each GTV in the plan was also observed to examine the suitability of a 1‐mm GTV–PTV margin for these cases.

### 2.4 Patient‐specific quality assurance

Patient‐specific QA was performed using the SRS MapCHECK. This device has 1013 N‐type silicon diodes, arranged in two slightly offset planar arrays to create a 7.7 × 7.7‐cm^2^ array with an inter‐detector spacing of 2.47 mm.^28^

The plans were copied onto a CT scan of the SRS MapCHECK inserted into the StereoPHAN with a 1‐mm slice thickness. Due to the limited active area of the SRS MapCHECK (7.7 × 7.7 cm^2^), two QA plans were required per introduced error to sample all high‐dose regions. For each pair of QA plans, the isocenter was shifted translationally within the TPS so that the detector intersected high‐dose regions. One plan sampled the three superior targets (PTV1, 2, and 3), and the other sampled PTV3, 4, and 5 (the inferior targets). The planned dose was recalculated for each of these QA plans for comparison with measured doses, again with a 1‐mm^3^ voxel size.

Dose measurements were taken with the SRS MapCHECK inserted into the StereoPHAN and arranged on the headboard using shaped foam as the MultiMet‐WL was (Figure 2). The phantom was aligned using the room lasers and markers on the phantom. Only the base plan was delivered and the measured dose was analyzed against the calculated doses for all QA plans with rotations included. This was done via gamma analysis that is described in detail by Low et al.,^29^ using SNC Patient software (v8.3, Sun Nuclear Corporation, Melbourne, Florida, USA).

## 3 RESULTS

### 3.1 Mechanical axes measurements

Monthly QA results for mechanical rotational axes were analyzed for seven months from January to July, 2021 for three Varian TrueBeam linacs available. Average variation in couch angle for all months and linacs was found to be (0.01 ± 0.03)°. Average gantry angle variation was (0.04 ± 0.06)° and collimator angle variation was (0.06 ± 0.08)°. Across all linacs, there was a negligible rotational error in all three axes, with the vast majority of measurements (95%) showing a variation of ≤0.1°; the largest discrepancy across all measurements had a magnitude of 0.3°.

### 3.2 Off‐axis Winston–Lutz test results

Each of the five targets and the isocenter were analyzed individually for the off‐axis WL test. The passing criteria of the off‐axis WL test are that discrepancy in position in each of the tested variables be <1 mm. These variables were the 3D position, the 2D position, and displacement in the *x*‐ and *y*‐position for each image. Failure in the 2D position is indicative of failure in either the *x*‐ or *y*‐direction and, therefore, was used as a comparative indicator in this study. Table 4 shows the maximum 2D difference across the eight images for each target. Values for the isocenter are acceptable across all introduced errors. Targets 4 and 5 failed for both couch and collimator rotations ≥0.5°. All targets failed for couch angle rotations ≥1.5°. The only targets to fail when a gantry angle rotation was applied were targets 2 and 4 at rotations of 1.0 and 1.5°, respectively.

**TABLE 4:** Maximum discrepancies in 2D position across eight images as calculated by the off‐axis WL test (mm)

	Rotation (°)	Isocenter	Target 1	Target 2	Target 3	Target 4	Target 5
**baseline**	0.0	0.48	0.66	0.69	0.63	0.91	0.59
**Couch**	0.5	0.65	0.65	0.51	0.90	**1.31**	**1.37**
	1.0	0.58	0.90	0.87	**1.14**	**1.82**	**1.98**
	1.5	0.57	**1.40**	**1.25**	**1.00**	**2.16**	**2.39**
	2.0	0.39	**1.84**	**1.74**	**2.30**	**2.64**	**2.88**
**Gantry**	0.5	0.41	0.38	0.84	0.39	0.91	0.53
	1.0	0.38	0.58	**1.16**	0.47	0.90	0.61
	1.5	0.51	0.57	**1.42**	0.52	**1.18**	0.61
	2.0	0.48	0.57	**1.69**	0.51	**1.46**	0.75
**Collimator**	0.5	0.55	0.84	0.97	0.93	**1.29**	**1.15**
	1.0	0.63	**1.11**	**1.49**	**1.14**	**1.83**	**1.98**
	1.5	0.61	**1.32**	**1.54**	**1.39**	**2.34**	**2.56**
	2.0	0.61	**1.39**	**1.73**	**1.51**	**2.56**	**2.75**

*Note*: Failing targets are shown in red and bold font.

Abbreviation: WL, Winston–Lutz.

### 3.3 GTV and PTV dose coverage

**FIGURE 3:**
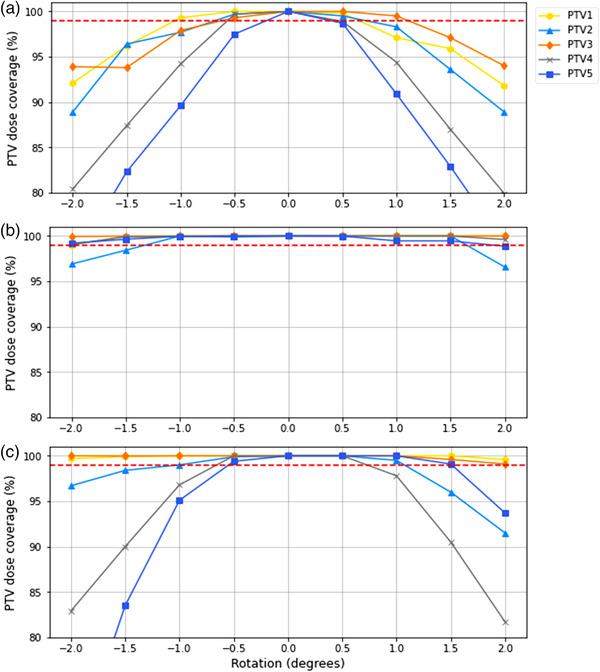
Percentage of each PTV covered by the prescription isodose line with rotations introduced to the (a) couch, (b) gantry, and (c) collimator angles. The red dashed line indicates the acceptable threshold of 99% PTV coverage. PTV, planning target volume

PTV coverage was calculated using the TPS for each of the plans created (Figure 3). The departmental protocol requires PTV coverage of the prescription isodose line for brain metastases to be ≥99%.

Errors in each of the rotational axes (gantry, couch, and collimator) were introduced independently. Greatest effects were seen for errors in couch angle, with a +0.5° error in couch angle resulting in the coverage of the two furthest off‐axis targets, (PTV4 and 5), to fall below 99%. There was some variation in results between the positive and negative rotations due to a nonuniform field; however, with a −0.5° couch rotation, PTV5 still fell below the threshold.

Comparatively, errors in gantry position had a much lesser impact on target coverage. All targets maintained acceptable coverage up until a gantry angle rotation of +2.0 or −1.5°.

For collimator angles, a rotation of ±0.5° saw negligible reduction in coverage; however, at ±1.0° some targets began to fall below 99% coverage. The most affected targets were the further off‐axis targets and the two offset from the superior/inferior axis.

**FIGURE 4:**
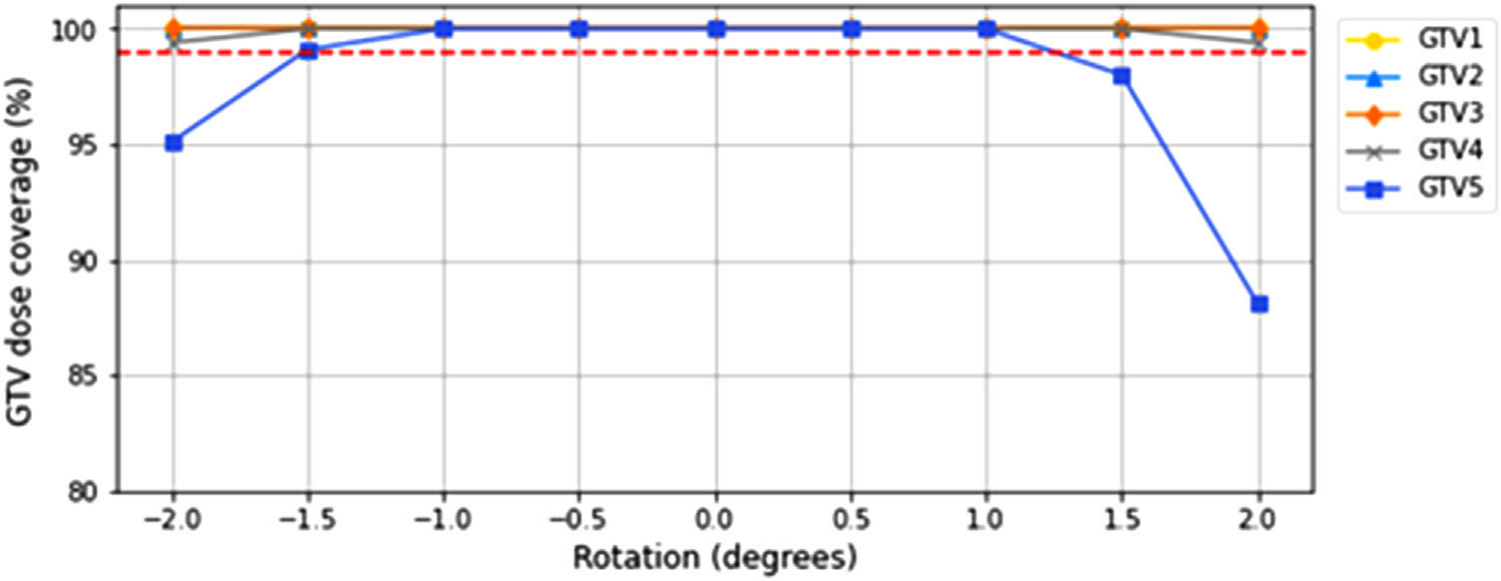
Percentage of each GTV covered by the prescription isodose line with rotations introduced to the couch angle. The red dashed line indicates 99% GTV coverage. GTV, gross tumor volume

As with PTV coverage, the greatest effects on GTV coverage were seen for errors in couch angle. Even so, the GTV coverage remained at 100% for most couch rotations and only dropped below 99% for GTV5 with a −1.5° rotation and a ±2.0° rotation (Figure 4). For rotations in gantry and collimator angles, all GTV coverage values remained at 100%.

### 3.4 Patient‐specific QA results

For every plan, gamma analysis was undertaken at passing criteria of 1‐mm distance to agreement and 3%, 4%, and 5% dose to agreement. Pass rates were averaged across each of the four fields for the two SRS MapCHECK measurements taken (superior and inferior targets), and maximum and minimum passing rates were recorded. The accepted tolerance by the departmental protocol is an average gamma pass rate of 95%, which was used as a pass/fail threshold in this study.

**FIGURE 5:**
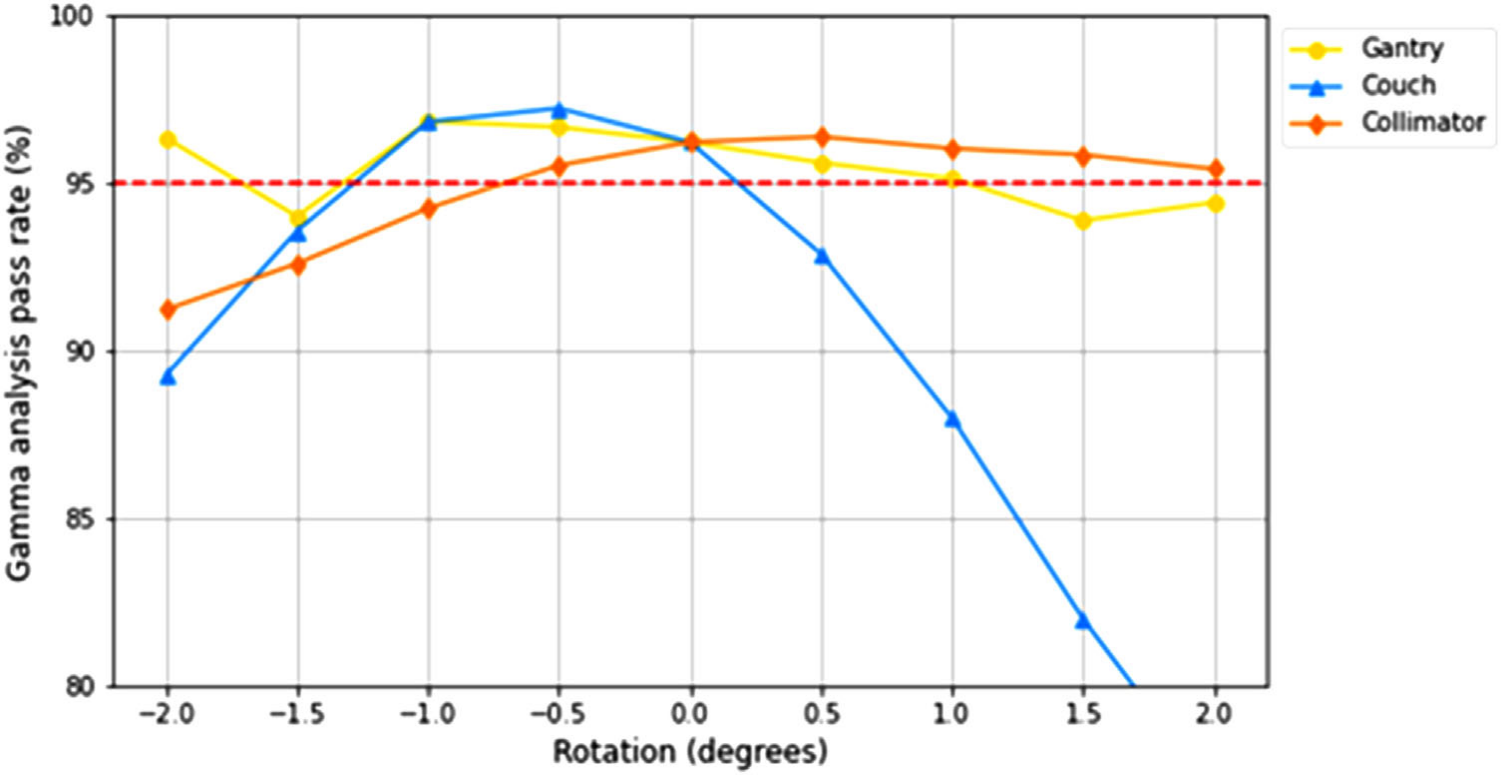
Gamma analysis pass rates in percentage for criterion of 5%/1 mm. The red dashed line represents the acceptable threshold of 95%

The resulting pass rates were asymmetrical across rotations in the positive and negative directions, as shown in Figure 5. For 5%/1 mm, positive direction rotations in the couch position dramatically reduced pass rates with an average of 92.9% at a +0.5° rotation. A −0.5° rotation had a much different effect with the pass rate increasing from the baseline 96.2% to 97.2%. Rotations in the gantry position had very little effect on gamma analysis pass rates with the average pass rate remaining above 95% for a ±1.0° rotation. The lowest average pass rate occurred for a +1.5° rotation at 93.9%; however, at −2.0° the pass rate increased to 96.3%.

For the collimator, rotations in the positive direction had little effect, whereas in the negative direction pass rates dropped below 95% at −1.0° and continued decreasing to 91.2% at 2.0°. For 4%/1 mm and 3%/1 mm, trends were almost identical to that of 5%/1 mm but with overall lower pass rates.

## 4 DISCUSSION

This study aimed to investigate the necessity of off‐axis WL testing with a consideration of whether patient‐specific measurement‐based QA is sensitive to rotational errors and a focus on maintaining GTV/PTV dose coverage. Although the off‐axis WL test was confirmed to detect rotational errors when or before PTV coverage became compromised, patient‐specific QA was shown to be inconsistent in the detection of rotational errors. Therefore, it has been recommended that the off‐axis WL test be conducted regularly.

The off‐axis WL test assesses the alignment of targets to high‐dose regions when both are off‐axis from the machine isocenter. When misalignment occurs via inaccuracy in gantry, couch, and collimator angle, the dose distribution will be impacted and hence target coverage.^16^, ^18^ The error in the couch angle was found to have the greatest impact upon both GTV and PTV dose coverage (Figure 3). For a rotation of +0.5° in couch angle, coverage for PTV4 and 5 was below the departmentally accepted threshold of 99%. For a 0.5° magnitude of error in gantry and collimator angles, coverage for all GTVs and PTVs remained above 99%. The observed qualitative trend of greater impact from couch angle error, as opposed to gantry and collimator errors, can be expected due to the couch rotation offsetting the dose delivered in the same direction for every angle of the gantry arc. In contrast, as the gantry rotates, the direction in which both the collimator and gantry are offset changes creating a blurring effect.

The magnitude of impact on coverage seen in this study is in alignment with other studies, including Nakano et al.^17^ that found for 1‐cm targets, at off‐axis distance of 3 cm, a 2.0° rotational error corresponded to a 9.1% decrease in target coverage. This off‐axis distance and size correspond to PTV1 and PTV3 of this study, which when using a ±2.0°rotation in couch angle saw a decrease in coverage of 8.0% and 6.0%, respectively (averaged between positive and negative rotations). This value is lower than the aforementioned study; however, this measurement only accounts for couch angle error as the mechanical axes were tested in isolation. This is a limitation of this study as clinically errors in multiple rotational axes simultaneously may occur with a compounded effect. Considering this, Nakano et al.^17^ applied rotations in *x*‐, *y*‐, and *z*‐axes simultaneously, which accounts for the small variation in results. Therefore, findings from this study relating to target coverage reflect expectations both qualitatively and quantitatively. However, a further limitation should be mentioned that this study did not investigate the impact that a high‐definition MLC may have compared to the Millennium MLC used in this study.

Documents such as the 2017 AAPM‐RSS practice guidelines for SRS/SBRT^30^ undergo thorough consensus processes and extensive review to develop minimum standards for safe practice. However, these guidelines do not include policy or standard operating procedures for departments, therefore specific procedures must be assessed per department. The aforementioned guidelines indicate tolerance levels for couch position indicators to be 0.5°. However, coverage for PTV4 and PTV5 fell below the departmentally accepted 99% for a couch angle error of this magnitude. Therefore, findings from this study indicate that this tolerance is inadequate to preserve 99% PTV coverage for the presented target distribution and a 0.5° couch angle tolerance may need to be reassessed. Alternatively, an off‐axis distance limit may be determined, which has been applied using several different methods in various studies,^13^, ^17^, ^18^, ^25^ or GTV–PTV margins modified as a function of off‐axis distance.

In this study, a GTV–PTV margin of 1 mm was applied as per department protocol, which is designed to account for errors in delivery. With this margin applied, despite the compromised PTV coverage observed, GTV coverage remained unaffected except for large couch angle errors (≥1.5°) (Figure 4). However, if a 0‐mm expansion was applied, the GTV coverage would experience the same reduction as observed for PTV coverage.

The impact of rotational errors on GTV/PTV coverage cannot be determined as a single value for all cases but is instead dependent on several factors, including target size and off‐axis distance.^16^, ^18^ A study by Nakano et al.^17^ determined off‐axis distance limits by evaluating coverage for a range of target sizes at a range of off‐axis distances and rotational errors. This is a time‐consuming approach that would need to be conducted by individual departments using specific protocols and tolerances. A different approach described by Roper et al.^18^ is to set acceptable coverage for a specific target and determine a maximum radius within which an isocenter can be located. This would be done for all targets creating a Venn diagram‐like overlapping region where the isocenter can be located. This approach will again require substantial work to develop for departments. In a study by Gao et al.,^13^ an off‐axis distance limit of 3 cm was determined for the tested equipment based on results of the off‐axis WL test. However, such a small off‐axis distance will limit target distributions suitable for SIMT SRS. Alternatively, reducing the accepted tolerance in couch angle will ensure better target coverage without limiting target distributions.

Through linear interpolation of the coverage results for the most affected target (PTV5, 7‐cm off‐axis) it was found that reducing couch angle error to 0.2° would preserve the departmentally acceptable 99% PTV coverage. Rotational errors have been found to have a greater impact on smaller targets at greater off‐axis distances^18^; therefore, this tolerance applies for ≥1‐cm targets up to 7‐cm off‐axis. The results of the routine QA of the linac‐mechanical axes at our center show that couch angle deviation is consistently ≤0.1°, suggesting a tightened tolerance of 0.2° is feasible for TrueBeam linear accelerators. Measured deviations of gantry and collimator angles are greater than that of couch angle, and on one occasion exceeded 0.2°. However, the impact of these errors is insignificant for target coverage even up to 0.5°, and, therefore, tolerance for collimator and gantry axes measurements could remain at 0.5°. Applying this tightened tolerance for couch angle will not require limiting off‐axis distance and, therefore, will have minimal effect on patient target distributions, which are suitable for SIMT SRS. This approach can also be supported by findings from this study without the need for additional and lengthy testing. For the safe delivery of treatments, not only do policies and standard operating procedures need to be developed and followed, but in the event, an error occurs this must be detected efficiently and consistently.

The widespread implementation of the off‐axis WL test is relatively recent, being introduced in 2016,^13^ and although several studies into purpose built phantoms and methodologies exist,^12^, ^13^, ^25^, ^26^ data on test frequency is currently unavailable. This study aimed to assess the necessity of routine off‐axis WL testing with the use of patient‐specific QA already in place.

In this study, the off‐axis WL test was shown to be consistently sensitive to rotational errors in couch, gantry, and collimator angles. The passing criteria of <1‐mm discrepancy in 2D location (Table 4) were consistently exceeded on or before the PTV coverage became compromised below acceptable limits (Figure 3). Studies have shown that for targets located at isocenter, rotational errors have limited dosimetric effect.^11^ Therefore, as expected, the target at isocenter met the passing criteria of the off‐axis WL test for all introduced errors. A couch angle rotation of 0.5° saw dose coverage for PTV4 and 5 fall below acceptable limits (Figure 3). Similarly, the off‐axis WL test failed for the corresponding targets at this value. Errors in gantry angles first saw dose coverage for PTV2 compromised at a magnitude of 1.5°, whereas the off‐axis WL test failed for this target at a 1.0° gantry angle rotation. For collimator angle errors, coverage was compromised at a magnitude of 1.0° for PTV2, 4, and 5. The off‐axis WL test detected the rotational error for targets corresponding to PTV4 and 5 at a magnitude of just 0.5°, and the target corresponding to PTV2 at 1.0° rotation. This study has, therefore, demonstrated that the off‐axis WL test is sensitive to detecting rotational errors before they affect departmentally accepted PTV coverage for this target geometry. If patient‐specific QA is capable of detecting these errors with similar sensitivity, the off‐axis WL test may be supplemented by this and, hence, be required less frequently.

The SRS MapCHECK has been validated by several studies for use in QA for SRS ^28^, ^31^, ^32^; however, specific application to rotational errors was yet to be tested. The SRS MapCHECK may hold some disadvantages in the detection of rotational errors over the purpose designed off‐axis WL test. In this study, the target geometry sampled by the SRS MapCHECK mimicked the target locations used in the off‐axis WL test, which are at points designed to detect rotational errors, however, in practice the SRS MapCHECK measures high‐dose regions of patient‐specific target geometries that may be less suited to this. In addition, each target in this study is not measured individually by the SRS MapCHECK, whereas the off‐axis WL test analyses targets individually. However, individual measurement of every target would become a time‐consuming process and, therefore, negate the aim of this study to increase QA efficiency.

Gamma analysis pass rates for QA using the SRS MapCHECK demonstrated inconsistencies regarding the detection of the introduced rotational errors at all sets of gamma analysis criteria; here, results for criteria 5%/1‐mm dose/distance to agreement will be discussed (Figure 5). A rotation of +0.5° in the couch angle resulted in an average gamma analysis pass rate of <95%, below the accepted tolerance used in this study. However, in the negative direction, a rotation of −1.5° was required before the average pass rate fell below this limit. At a rotation of −0.5°, the pass rate increased above the baseline value of 96.2% suggesting a small setup error of the SRS MapCHECK of 0.5°. However, even when taking this into account, PTV coverage was compromised for couch rotations of just 0.5°, and, therefore, if the measurement‐based QA was sensitive to these errors, only one measurement should have met the gamma analysis passing criteria that did not occur. For errors in the gantry angle, both positive and negative rotations of 1.5° magnitude saw the gamma analysis average pass rate drop below 95%. This is the magnitude at which target coverage was first compromised for gantry rotations that would indicate QA using the SRS MapCHECK to be sensitive enough to detect rotational errors of this kind. However, this is not the case as at −2.0° gantry rotation the average gamma analysis pass rate increased to above the 95% limit used in this study despite target coverage continuing to decrease. Similarly, QA results showed inconsistencies in the detection of rotational errors in the collimator angle. Average gamma analysis pass rates were <95% for negative collimator angle rotations of magnitude ≥1.0°, yet remained above this limit for all positive rotations.

The results of this study show, for the tolerance levels and target distributions used, patient‐specific QA with an SRS MapCHECK is inconsistent in detecting rotational errors. There was a significant impact of the rotational errors tested on the PTV coverage that may impact treatment efficacy. However, GTV dose coverage remained unaffected for both collimator and gantry angle errors and only fell for couch rotations of ≥1.5°. Routine monthly machine QA includes mechanical axes testing designed to detect rotational errors in couch, gantry, and collimator angles with results presented here showing consistently low levels of deviation. These tests confirm the coincidence of angle indicators and actual axis positions; however, the off‐axis WL test specifically assesses the alignment of off‐axis positions and the radiation field at those positions. In addition, routine machine QA assesses rotational axes in isolation, whereas the off‐axis WL test measures the cumulative effect of each rotation. Therefore, it is recommended from this study that the off‐axis WL test, which was shown to be sensitive to rotational errors of magnitudes that compromise PTV coverage, should be conducted at a regular interval to supplement the routine monthly QA. The frequency of this interval should be determined by individual departments, within the context of the GTV–PTV margins used, limitations on target off‐axis distances, and their routine mechanical QA results for that particular linac.

## 5 CONCLUSION

This study aimed to determine if QA could be streamlined with patient‐specific measurement‐based QA to supplement off‐axis WL testing, therefore reducing the frequency of the latter. Significant effects on PTV coverage were observed with rotational errors, especially in the couch angle, indicating that careful consideration of tolerance levels is required. With the use of a 0‐mm GTV–PTV expansion margin, this dose compromise will also apply to GTV coverage. However, with a 1‐mm margin, GTV coverage may be preserved with results here showing that GTV coverage remained at 100% except for with couch rotations of ≥1.5°. The off‐axis WL test was found to be sensitive to rotational errors with results consistently exceeding acceptable values when or before coverage fell to below the departmentally accepted limits. Although measurement‐based QA using the SRS MapCHECK has been previously confirmed to be accurate for SRS, these results showed inconsistency in the detection of rotational errors. It is recommended that the off‐axis WL test be conducted regularly as it is sensitive to errors that routine patient‐specific QA may not detect.

## CONFLICT OF INTEREST

The authors declare that there is no conflict of interest that could be perceived as prejudicing the impartiality of the research reported.

## AUTHOR CONTRIBUTIONS

Lauren M. M. Pudsey conducted project methods and wrote the manuscript with the guidance and assistance of other authors. Anatoly Rosenfeld and Giordano Biasi provided guidance as supervisors of the project. Anna Ralston informed project direction and manuscript revision. Joel Poder was responsible for the overall idea of the project and assisted with the development of methods and analysis of results. All authors were involved in a review of the manuscript.

## Data Availability

The data that support the findings of this study are available from the corresponding author upon reasonable request.
